# Olanzapine as an Adjunct in the Management of Refractory Psychogenic Excoriation With Comorbid Schizophrenia: A Case Report

**DOI:** 10.7759/cureus.8772

**Published:** 2020-06-22

**Authors:** Violet Yeager, Matthew Bogoyas, Bishoy Goubran, Lisandra Gonzalez, Gerardo F Ferrer

**Affiliations:** 1 Psychiatry, Larkin Community Hospital, Miami, USA; 2 Division of Clinical and Translational Research, Larkin Community Hospital, Miami, USA

**Keywords:** schizophrenia, psychogenic excoriation, long acting injectable, olanzapine, haldol decanoate

## Abstract

Neurotic or psychogenic excoriation (PE) is one of the most commonly diagnosed skin disorders associated with a primary psychiatric condition. PE is characterized by excessive picking and scratching of normal-appearing skin, and is often comorbid or is an inherent manifestation of affective disturbance and psychosis itself in schizophrenia. Evidence in the literature has demonstrated the therapeutic efficacy of selective serotonin reuptake inhibitors (SSRI) in treating PE. Other pharmacological treatments that have shown therapeutic benefits in case reports include doxepin, clomipramine, naltrexone, pimozide, and olanzapine. However, using adjunct therapeutic methods or augmentation in the treatment of neurogenic excoriation in the setting of schizophrenia is still not well explored. In this study, we discuss the case of a 59-year-old medically complex paraplegic male with schizophrenia comorbid with severe refractory PE. The patient had poor adherence to psychopharmacological treatment. Consequently, the patient was repeatedly hospitalized due to acute exacerbations of schizophrenic episodes and self-mutilation due to PE. After several failed treatment approaches, olanzapine 10 mg PO BID was added as an adjunct therapy to the Haldol® Decanoate (Janssen Pharmaceutica, Beerse, Belgium) at a dosage of 100 mg/month intramuscularly to control the acute PE symptoms. This treatment modality proved successful in this case, and the patient has been free from PE relapse for over one year of close follow-up. Olanzapine along with Haldol Decanoate long-acting injectable (LAI), might, therefore, be a useful adjunct therapeutic modality for patients with refractory PE with a comorbid diagnosis of schizophrenia and warrants further research.

## Introduction

Psychogenic excoriation (PE) is subcategorized under the umbrella of obsessive-compulsive and related disorders in the Diagnostic and Statistical Manual of Mental Disorders, 5th Edition (DSM-5) [1]. Its diagnostic criteria include recurrent skin-picking, resulting in skin lesions, and repeated attempts to decrease or stop skin-picking, which causes clinically significant distress or impaired functioning. Another criterion is that the skin-picking cannot be attributed to the physiologic effects of a substance (e.g., cocaine) or another medical condition (e.g., scabies) [2]. Emerging studies have shown difficulty in determining whether PE is diagnostically distinct from related disorders such as obsessive-compulsive disorder, schizophrenia, or a coexisting comorbid separate diagnosis [3,4]. Simultaneously, comorbid depression, mood, and anxiety disorders have shown a high prevalence of excoriation disorder along with body dysmorphic disorders and obsessive-compulsive disorders [5-7]. 

The mainstream treatment options for PE lack a general consensus but include behavioral therapy and habit reversal therapy; however, they primarily involve medications such as selective serotonin reuptake inhibitors (SSRI) [8] or N-acetyl cysteine [9]. Also, other pharmacological modalities including doxepin, clomipramine, naltrexone, pimozide, and olanzapine have shown some success [10]. Moreover, the use of second-generation antipsychotics either as monotherapy or as an adjunct with SSRI/serotonin-norepinephrine reuptake inhibitors (SNRI) has also been explored [11,12]. However, limited information is available on augmentation treatment options for refractory PEs associated with psychiatric comorbidities, which can complicate treatment for conditions such as schizophrenia. Further, to the best of our knowledge, no studies have explored utilizing olanzapine as an adjunct to Haldol® Decanoate (Janssen Pharmaceutica, Beerse, Belgium) long-acting injectable (LAI) in the treatment of these patients. Patients with schizophrenia commonly require LAI antipsychotics to prevent relapse, recurrence, and to tackle low medication adherence and poor compliance [13,14]. Therefore, we believe that it is important to highlight the possible use of adjunct medications in this specific population with comorbid PE. In this report, we discuss the case of a patient diagnosed with schizophrenia and comorbid severe refractory excoriation disorder who was treated successfully by adding olanzapine as an adjunct to LAI first-generation antipsychotic Haldol Decanoate. This treatment modality has shown good results, and the patient has gone into remission of PE episodes for a duration of over a year.

## Case presentation

A 59-year-old Asian male with a past psychiatric history of schizophrenia was admitted initially to the inpatient behavioral unit for an acute exacerbation of psychotic symptoms. His past psychiatric history was pertinent for recurrent PE episodes with severe self-mutation from skin-picking of which some episodes coincided with psychotic episodes for over a year. On admission, the patient was on several psychotropic medications and sedatives including alprazolam, valproic acid, quetiapine, and trazodone. However, the patient continued to experience multiple recurrent episodes of severe PE. Moreover, the patient had multiple comorbid medical conditions that included seizures, congestive heart failure, hypertension, diabetes mellitus, hypothyroidism, and paraplegia, which required additional pharmacological treatments. The patient was intermittently compliant with taking his medications.

**Figure 1:**
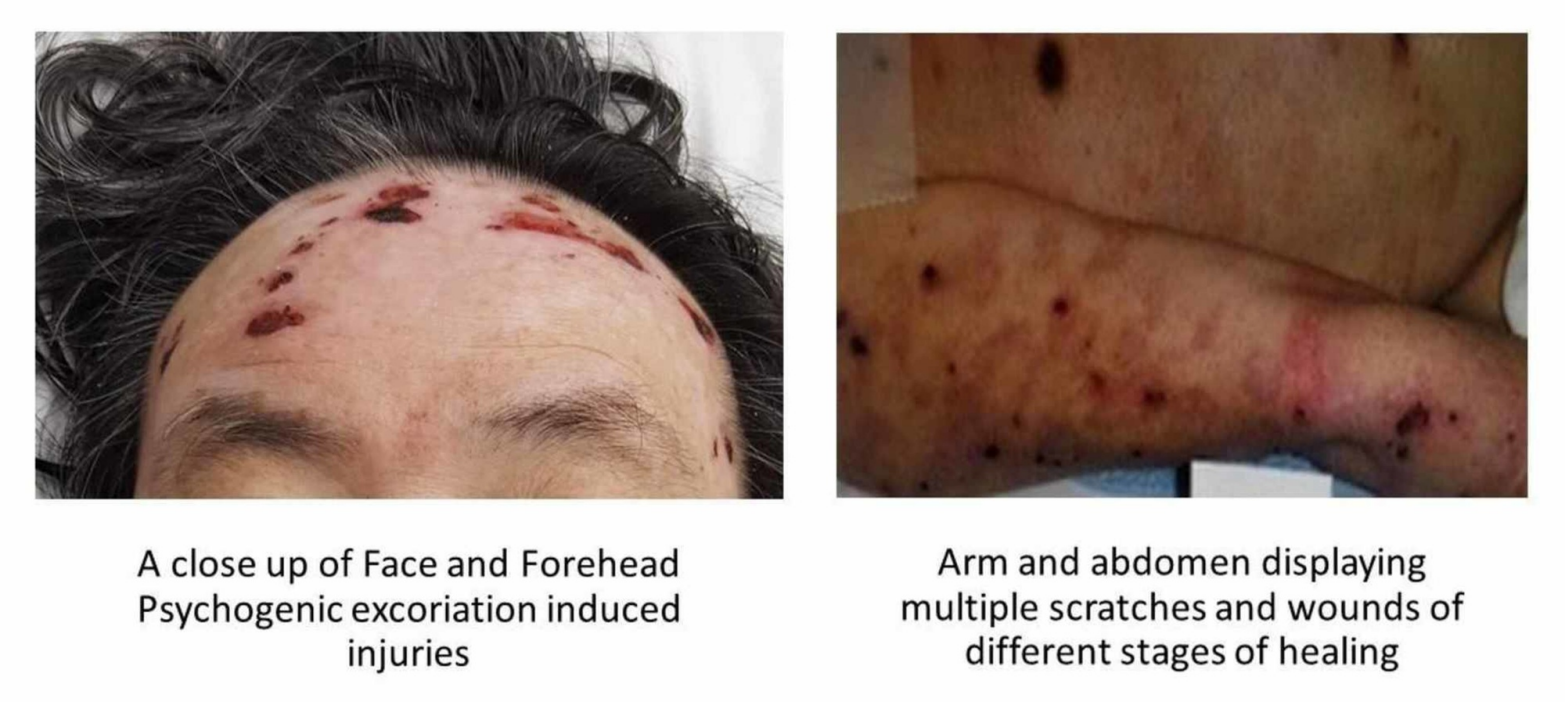
Severe refractory psychogenic excoriation - self-inflicted wounds

The patient had undergone several failed attempts in the past to treat the PE including utilizing SSRI; however, he continued to experience PE, as reported via collateral information. On admission, the patient was grossly disorganized, with auditory hallucinations along with multiple areas of self-mutilation including the face, arms, and abdomen due to skin-picking (Figure 1).

On admission, the patient was medically considered for alternative causes of the skin manifestations, and dermatology was consulted. The patient also had a negative urine toxicology report with collateral information supporting abstinence from illicit drugs. Per collateral information, the patient was diagnosed with schizophrenia and PE at distinct intervals. Upon admission and after a thorough assessment, the patient was diagnosed with schizophrenia, multiple episodes, and refractory PE.

The patient's psychopharmacological treatment was challenging due to the myriad medical comorbidities and lack of compliance, which required tailoring of management. The patient required long-term care. Throughout the course of care, the patient was managed for the schizophrenic episode and PE with the following atypical oral antipsychotic monotherapies: quetiapine 500 mg/d, olanzapine 10 mg/d, and combination therapies of antipsychotics plus mood stabilizers valproic acid 1000 mg/d and carbamazepine 400 mg/d in the hospital. Although these pharmacotherapies achieved temporary remission of a schizophrenic episode, it was only partially effective towards the PE. His hallucinations and disorganized thought content often went into remission while his PE persisted.

**Figure 2:**
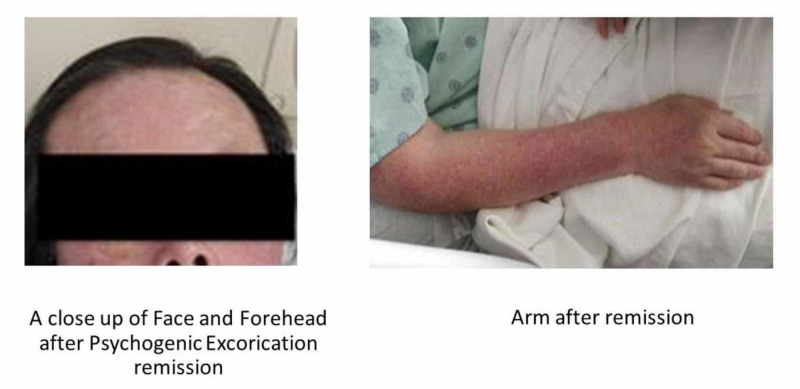
Patient after remission of psychogenic excoriation

The patient was subsequently started on Haldol 5 mg PO TID to further manage the schizophrenic symptoms. However, the patient was only intermittently adherent to medication, which led to psychotic relapses. Consequently, Haldol Decanoate 100 mg monthly injection was started to manage non-compliance and to provide long-term coverage. The LAI resulted in improvement in the patient's condition and reduced schizophrenic episodes and severity. However, the PE did not improve with this adjustment. After explaining to the patient that olanzapine was an off-label therapy for PE, we obtained consent to provide this treatment. Olanzapine 10 mg PO BID was then added to the treatment with Haldol Decanoate, which resulted in the remission of excoriations. Over more than 12 months, the patient has been maintained on long-acting Haldol Decanoate 100 mg monthly injection and olanzapine 10 mg BID while in long-term care at the hospital with close monitoring. No symptoms or adverse events have occurred. The patient’s PE has resolved without relapses (Figure 2).

## Discussion

We reported a case of a 59-year-old male with recurrent, treatment-resistant PE episodes with severe self-mutation from skin-picking of which many coincided with episodes of schizophrenic exacerbation. Skin-picking, in this case, was very severe and visible on the face, back, and thighs. The patient’s PE was refractory to multiple medication trials whether with antipsychotics as monotherapy or in combination with SSRI. The patient was selectively compliant given his medical complexity and a large number of daily medications. This led to multiple recurrences and exacerbation of his schizophrenia episodes. It was, therefore, the decision of the interdisciplinary medical team to utilize an LAI. Accordingly, treatment with 100 mg Haldol Decanoate was used to stabilize the patient’s schizophrenic episodes. While our patient did have an attenuation of his psychotic symptoms, his PE behavior was still not controlled and an augmentation trial with a second-generation antipsychotic was warranted. In this case, olanzapine 10 mg BID was used as an adjunct therapy to control his excoriation. This case report demonstrates the efficacy of olanzapine as a successful adjunct therapy to Haldol Decanoate in the management of refractory PE with comorbid schizophrenia.

Previous studies have elucidated that PE can be managed by SSRIs [15] and dopamine-blocking psychotropic drugs. A comprehensive treatment regimen utilizes both psychotropic drugs, cognitive-behavioral therapy (CBT), and therapeutic modalities for skin-picking. Augmentation has been explored and, for instance, olanzapine has been studied in conjunction with SSRI [16]. Moreover, utilizing aripiprazole as an adjunct for augmenting venlafaxine has shown promising results [11]. Haloperidol has also been studied in conjunction with fluvoxamine [17]; however, to our knowledge, there are no rigorous studies yet that have investigated the efficacy of Haldol Decanoate LAI combined with olanzapine in the management of PE in the context of schizophrenia.

In our case, this treatment modality has brought remission to our patient's PE, and his inflicted wounds, scratches, and skin conditions have improved significantly. The patient’s schizophrenia and selective compliance have been managed with the LAI. Our study, therefore, demonstrates the utility of adjunct olanzapine in patients with PE in the setting of schizophrenia, while providing maintenance treatment of comorbid psychiatric conditions and medical conditions as observed in the present case.

## Conclusions

Given that refractory PE may present with multiple psychiatric and medical comorbidities, olanzapine may be an effective adjunct option to Haldol Decanoate LAI in the treatment of these patients. Additionally, LAI antipsychotics with adjunct olanzapine may be beneficial in cases of non-compliance in patients with refractory PE in the context of Schizophrenia. Further clinical research and trials are required to validate our findings and investigate the benefits of utilizing olanzapine along with other forms of LAI antipsychotic medications.
